# Accessibility and Openness to Diabetes Management Support With Mobile Phones: Survey Study of People With Type 1 Diabetes Using Advanced Diabetes Technologies

**DOI:** 10.2196/36140

**Published:** 2022-06-24

**Authors:** Yu Kuei Lin, Caroline Richardson, Iulia Dobrin, Rodica Pop-Busui, Gretchen Piatt, John Piette

**Affiliations:** 1 Department of Internal Medicine University of Michigan Medical School Ann Arbor, MI United States; 2 Department of Family Medicine University of Michigan Medical School Ann Arbor, MI United States; 3 Department of Learning Health Sciences University of Michigan Medical School Ann Arbor, MI United States; 4 Veterans Affairs Ann Arbor Healthcare System Center for Clinical Management Research Ann Arbor, MI United States; 5 Department of Health Behavior and Health Education University of Michigan School of Public Health Ann Arbor, MI United States

**Keywords:** type 1 diabetes, diabetes technology, diabetes self-management, diabetes, self-management, cross-sectional, glucose monitor, insulin pump, mHealth, mobile health, access, acceptability, feasibility, cell phone, text message, smartphone, cellphone, mobile device, patient communication, interactive voice response call, glycemic control

## Abstract

**Background:**

Little is known about the feasibility of mobile health (mHealth) support among people with type 1 diabetes (T1D) using advanced diabetes technologies including continuous glucose monitoring (CGM) systems and hybrid closed-loop insulin pumps (HCLs).

**Objective:**

This study aims to evaluate patient access and openness to receiving mHealth diabetes support in people with T1D using CGM systems or HCLs.

**Methods:**

We conducted a cross-sectional survey among patients with T1D using CGM systems or HCLs managed in an academic medical center. Participants reported information regarding their mobile device use; cellular call, SMS text message, or internet connectivity; and openness to various channels of mHealth communication (smartphone apps, SMS text messages, and interactive voice response [IVR] calls). Participants’ demographic characteristics and CGM data were collected from medical records. The analyses focused on differences in openness to mHealth and mHealth communication channels across groups defined by demographic variables and measures of glycemic control.

**Results:**

Among all participants (N=310; female: n=198, 63.9%; mean age 45, SD 16 years), 98.1% (n=304) reported active cellphone use and 80% (n=248) were receptive to receiving mHealth support to improve glucose control. Among participants receptive to mHealth support, 98% (243/248) were willing to share CGM glucose data for mHealth diabetes self-care assistance. Most (176/248, 71%) were open to receiving messages via apps, 56% (139/248) were open to SMS text messages, and 12.1% (30/248) were open to IVR calls. Older participants were more likely to prefer SMS text messages (*P*=.009) and IVR calls (*P*=.03) than younger participants.

**Conclusions:**

Most people with T1D who use advanced diabetes technologies have access to cell phones and are receptive to receiving mHealth support to improve diabetes control.

## Introduction

About 1.6 million people in the United States have type 1 diabetes (T1D) [1], and the prevalence continues to increase both in the United States [2] and globally [3]. Managing T1D requires comprehensive skill sets from patients and care providers including proficiency in monitoring and interpreting glucose levels, and administering appropriate doses of insulin based on a range of variables including carbohydrate intake, glucose levels, physical activity, medications, stress, illness, and recent hypoglycemic episodes [4].

Technologies, such as continuous glucose monitoring (CGM) systems and hybrid closed-loop insulin pumps (HCLs), can provide patients with T1D with real-time glucose information and algorithm-based insulin delivery [5]. CGM systems are now considered the standard of care for people with T1D [5], and the number of people using CGM systems has increased rapidly [6]. However, a significant proportion of CGM and HCL users fail to achieve optimal glucose targets [7,8] based on evidence from both clinical trials [9-13] and real-world observational studies [14-16]. Additional support for individuals with T1D beyond these technologies may be critical to optimize diabetes control and minimize complications [17].

More than 85% of the US [18,19] and 48% of the global population [20] uses a smartphone, and nearly half of US smartphone users use their mobile devices to access information and track progress on health-related goals [19]. Health support via mobile devices (ie, mobile health [mHealth]) thus offers a great opportunity to improve access to effective behavioral interventions [21]. The field of mHealth includes a variety of digital tools and communication channels, including smartphone apps, SMS text messages, and interactive voice response (IVR) calls to deliver information and behavior change support [22]. Studies demonstrate that these digital aids can improve patient diabetes knowledge and reduce hyperglycemia [23-25] through digitalized diabetes education, enhanced communications, and incorporations of patient-generated data [23,26]. In 2020, an international collaborative published a consensus on future directions in diabetes mHealth, including diversifying interventions to meet the needs of heterogeneous diabetes populations [21]. Other frameworks for further enhancing technology-enabled diabetes care emphasized the significance of data-driven, two-way feedback loops [27,28] for personalizing and targeting programs that improve T1D self-management.

Given that CGM systems provide data about glucose levels in real time, opportunities exist for the development of T1D mHealth support programs that retrieve data continuously and use that information to deliver timely and personalized patient feedback [27]. However, little is known about mobile phone use among people with T1D using advanced diabetes technologies. In addition, people’s receptivity to mHealth programs may vary according to their demographic characteristics and glycemic control, and some patients may not be comfortable sharing CGM data with mHealth platforms. Finally, there is a lack of information on people’s relative openness to various communication channels including smartphone apps, SMS text messages, and IVR calls.

To address these gaps in knowledge, we conducted a survey among a large sample of individuals with T1D using CGM systems and receiving diabetes care in an academic medical center. Here we report the findings from that survey including information about participants’ access to mobile technology; receptivity to mHealth interventions that require sharing their CGM data; and openness to communication via stand-alone apps, SMS text messaging, or IVR calls.

## Methods

### Ethics Approval

The survey was conducted between January and April 2021 after receiving approval from the University of Michigan Institutional Review Board (HUM00189672). The sampling frame for the survey was the population of adults with T1D receiving care through outpatient clinics associated with the University of Michigan Health System.

### Setting and Recruitment

The University of Michigan Health is a tertiary health center that provides health care to the surrounding communities, with more than 1 million people living in southeastern Michigan, and regularly supports diabetes care for about 3000 adults with T1D. A total of 1024 adults with diagnoses of T1D and ongoing CGM use were identified from the electronic medical record (EMR) system and invited via emails sent through REDCap. Candidates with missing or invalid email addresses were contacted via postal letters and telephone calls. The investigators avoided directly contacting their own patients for recruitment to prevent possible coercion or sampling biases. Survey participants provided written informed consent for linkage of their surveys with demographic data from the EMR and glucose data from their CGM systems. All people determined to be aged ≥18 years, have T1D, and use CGM systems based on EMRs were included in the study and analyses. Participants without 4-week CGM data within the past 3 months were excluded from the analyses involving CGM data.

### Survey Measures

The survey assessed participants’ durations of diabetes, CGM type and use duration, and insulin pump use information. Cellphone use, including the frequency of the participant carrying the cellphone (“How often do you have your cellphone with you?”) and cellular connectivity for calls and SMS text messages (“How often does your cellphone have good reception for text messages or phone calls?”), and internet access (“How often does your cellphone have access to the internet?”) at home, at work, and outside of home and work were assessed. Items developed for the study asked about participants’ receptivity to mHealth diabetes interventions and openness to different mHealth communication channels. Specifically, we asked “Cellphones could be used for receiving on-site, real-time support as we often carry them around...If you could get additional support at the time of high or low glucose levels to help you with your glucose control, which method(s) would you prefer?” (The response options were apps, SMS text messages, IVR calls, and “do not want diabetes support delivered through cellphone.”) Participants could select more than one communication channel option as their response. Surveys also assessed participants’ willingness to share real-time CGM information for glucose control support. Participants were encouraged to complete the survey directly via REDCap. Study team members conducted telephone surveys for participants without immediate access to the internet.

### EMRs Review and CGM Data Collection

Participants’ age, sex, race, ethnicity, and hemoglobin A_1c_ (HbA_1c_) levels were abstracted from the EMR. Recent CGM data [29] (ie, within 3 months prior to survey completion) were abstracted from CGM glucose reports uploaded to EMRs or directly from participants with CGM glucose information portals [30,31]. CGM data were collected for 4 weeks for the following measures: percent of time using a CGM system, average glucose level, percent of time spent with glucose levels above 180 (time above range [TAR]) and above 250 mg/dL, and percent of time below 70 (time below range [TBR]) and below 54 (50 for Medtronic CGM system) mg/dL [8].

### Statistical Analysis

Using the Cochran formula, we calculated that a sample of 280 respondents was needed to determine the prevalence of people receptive to mHealth diabetes interventions at a 95% confidence level with 5% precision for a pool of 1024 potential respondents. We conducted descriptive analyses of participants’ demographics characteristics and CGM glucose data. The Mann-Whitney *U* test was used to evaluate the difference in age and HbA_1c_ levels between participants and nonrespondents; differences in age, diabetes duration, and CGM glucose information between participants who were versus were not receptive to receiving mHealth interventions; differences in patient characteristics of respondents who were open to receiving mHealth support through various communication channels; and differences in TAR and TBR between female and male participants. Logistic regression analysis was used to evaluate sex differences between participants receptive versus unreceptive to receiving mHealth support and open to various communication channels for receiving mHealth support. For the analyses evaluating the characteristics of respondents open to various communication channels (ie, app vs SMS text message, app vs IVR calls, and SMS text messages vs IVR calls), participants who selected both communication channels were excluded from the analyses. *P*<.05 was considered to be statistically significant.

## Results

### Participant Characteristics

**Figure 1:**
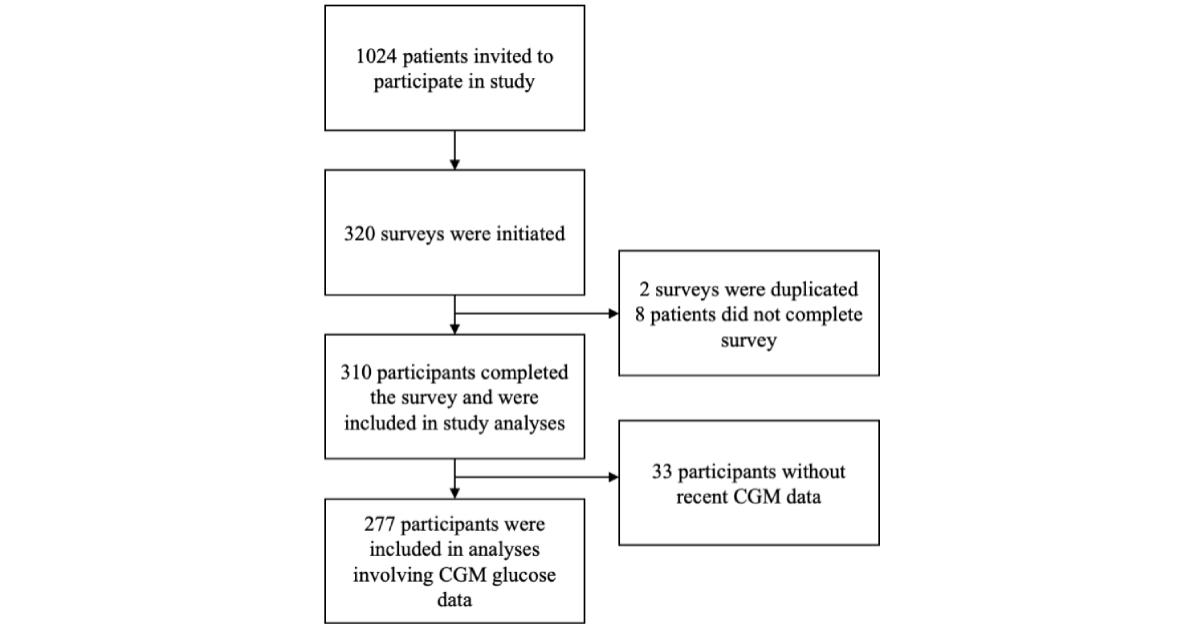
Patient participation flowchart. CGM: continuous glucose monitoring.

A total of 310 eligible participants completed the survey (Table 1), and 4-week CGM data within the last 3 months were successfully collected from 277 (89.4%) participants (Figure 1). There was no significant difference in age or HbA_1c_ levels between participants and other contacted candidates who did not complete the survey. A higher proportion of responders were female (n=198, 63.9%) compared to nonrespondents (360/714, 50.4%). No significant differences in TAR and TBR were identified between female and male participants.

**Table 1:** Participant demographics (N=310).

Characteristics			Participants
**Sex, n (%)**			
	Female	198 (63.9)	
	Male	112 (36.1)	
Age (years), mean (SD)			45 (16)
Age (years), median (IQR)			43 (31-58)
**Race, n (%)**			
	White or Caucasian	289 (93.2)	
	Black or African American	10 (3.2)	
	Asian	3 (1.0)	
	Refused to answer/unknown	1 (0.3)	
	Other	7 (2.3)	
**Ethnicity, n (%)**			
	Non-Hispanic	295 (95.2)	
	Hispanic	9 (2.9)	
	Refused to answer/unknown	6 (1.9)	
Duration of diabetes (years), median (IQR)			23 (14-32)
**Duration of CGM^a^ use, n (%)**			
	0-3 months	9 (2.9)	
	4-6 months	13 (4.2)	
	7-12 months	23 (7.4)	
	1 year to 3 years	131 (42.3)	
	4-6 years	80 (25.8)	
	>6 years	54 (17.4)	
**CGM model, n (%)**			
	Dexcom G5	4 (1.3)	
	Dexcom G6	277 (89.4)	
	Medtronic Guardian Sensor 3	29 (9.4)	
**Using insulin pump, n (%)**			245 (79.0)
	With auto-suspension features	164 (52.9)	
	With closed-loop features	149 (48.1)	
Last HbA_1c_^b^ level (%), median (IQR)			7.2 (6.5-7.8)
Time of CGM use (%), median (IQR)			97 (88-99)
CGM average glucose level (mg/dL), median (IQR)			159 (143-178)
TAR^c^ on CGM (%), median (IQR)			32 (20-44)
TBR^d^ on CGM (%), median (IQR)			1.4 (0.6-3.0)

^a^CGM: continuous glucose monitoring.

^b^HBA_1c_: hemoglobin A_1c_

^c^TAR: time above range.

^d^TBR: time below range.

### Access to mHealth

Of all 310 participants, 304 (98.1%) reported using cellphones. All these individuals reported using a smartphone, with 68.1% (207/304) using an iPhone and 29.9% (91/304) using an Android phone. About 90.1% (274/304) of participants reported carrying their mobile devices with them all or most of the time and that their mobile devices have connectivity for phone calls, SMS text messages, and the internet all or most of the time (Table 2). Participants were least likely to have their phones with them while at work and were least likely to have internet access when outside of home and work.

**Table 2:** Accessibility to mobile health support (N=310).

		Having cellphone accompanied, n (%)		Good reception for phone calls or SMS text messages, n (%)		Having access to the internet, n (%)
**At home**						
	All the time	185 (59.7)	187 (60.3)		226 (72.9)	
	Most of the time	105 (33.9)	109 (35.2)		68 (21.9)	
	About half of the time	12 (3.9)	9 (2.9)		9 (2.9)	
	Less than half of the time	4 (1.3)	3 (1.0)		4 (1.3)	
	Rarely	4 (1.3)	2 (0.6)		3 (1.0)	
**At work**						
	All the time	195 (62.9)	170 (54.8)		217 (70.0)	
	Most of the time	81 (26.1)	116 (37.4)		68 (21.9)	
	About half of the time	8 (2.6)	16 (5.2)		15 (4.8)	
	Less than half of the time	9 (2.9)	2 (0.6)		1 (0.3)	
	Rarely	17 (5.5)	6 (1.9)		9 (2.9)	
**Outside of home and work**						
	All the time	225 (72.6)	114 (36.8)		121 (39.0)	
	Most of the time	73 (23.5)	183 (59.0)		145 (46.8)	
	About half of the time	8 (2.6)	9 (2.9)		22 (7.1)	
	Less than half of the time	4 (1.3)	3 (1.0)		15 (4.8)	
	Rarely	0 (0.0)	1 (0.3)		7 (2.3)	

### Receptivity to mHealth Support

Of the 310 participants, 248 (80%) were receptive to receiving diabetes self-care support through their phones with the goal of improving their glucose control. There were no significant differences in sex, age, diabetes duration, average glucose level, TAR, TBR, and the percent of time spent with glucose levels above 250 mg/dL and below 54 mg/dL between those who were versus were not receptive to receiving mHealth support. Among participants receptive to mHealth support, 98% (243/248) responded that they would “very much” or “probably” be willing to share real-time glucose level data to receive tailored support for diabetes management.

### Openness to Various Communication Channels for Receiving mHealth Support

Among those who were receptive to mHealth support, 71% (176/248) were open to receiving support via apps, 56% (139/248) were open to SMS text messages, and 12.1% (30/248) were open to IVR calls. Participants open to apps but not IVR calls were younger than those open to IVR calls but not apps (Table 3). Similarly, participants open to apps but not SMS text messages were younger than those open to SMS text messages but not apps. No significant differences in diabetes duration, average glucose level, TAR, TBR, and time spent above 250 mg/dL or below 54 mg/dL were observed between those who were open to receiving diabetes support through apps, SMS text messages, or IVR calls. We also observed no sex differences in those open to various communication channels.

**Table 3:** Patient demographics and glycemic characteristics grouped by openness to mobile health communication channels.

	Apps, median (IQR)	SMS text messages, median (IQR)	IVR^a^ calls, median (IQR)	*P* value^b^		
				Apps vs SMS text messages^c^	Apps vs IVR calls^c^	SMS text messages vs IVR calls^c^
Age (years)	40 (28-54)	44 (32-58)	53 (36-64)	.009	.03	.12
Duration of diabetes (years)	24 (14-32)	23 (12-32)	21 (15-40)	.45	.98	.05
Average glucose level (mg/dL)	158 (143-175)	157 (141-176)	153 (145-182)	.88	.57	.99
TAR^d^ (%)	30 (19-42)	31 (18-43)	30 (20-45)	.99	.50	.79
Time with glucose level >250 mg/dL (%)	7 (2-13)	7 (2-13)	5 (2-14)	.98	.95	.69
TBR^e^ (%)	1.4 (0.5-3.0)	1.5 (0.7-3.0)	2.4 (0.8-3.8)	.51	.90	.05
Time with glucose level <54 mg/dL (%)	0.2 (0-0.6)	0.2 (0-0.5)	0.2 (0-0.7)	.58	.13	.44

^a^IVR: interactive voice response.

^b^Statistical analysis conducted with the Mann-Whitney *U* test.

^c^Participants who selected both communication channels were excluded from the analysis.

^d^TAR: time above range.

^e^TBR: time below range.

## Discussion

### Principal Findings

In this survey of a large sample of people with T1D who used CGM systems and HCLs, nearly all participants used smartphones, and nearly all reported the ability to make phone calls, receive SMS text messages, and connect to the internet most of the time. Participants were receptive to receiving support for diabetes care, including being willing to share CGM data automatically so that mHealth support could be personalized based on their clinical needs. When asked about their openness to various communication channels for receiving mHealth support, the majority were open to apps or SMS text messaging, and only a smaller proportion of individuals indicated openness to receiving IVR calls. Older participants preferred to receive mHealth support through SMS text messaging or IVR calls over apps.

### Comparison to Prior Work and Implications for Future Research

Prior studies have shown that adolescents with T1D are receptive to self-management assistance via mHealth tools [32]. This study adds to this body of evidence on the accessibility and receptivity to using mHealth interventions among adults with T1D who use advanced diabetes technologies to monitor their glycemic control and manage insulin administration. mHealth tools have the capability of providing two-way communication for effective interventions [27]. Advances in these apps have used artificial intelligence (AI) and adaptive messages based on individuals’ status [33] to further personalize the support and target individuals’ ongoing needs. Given that in this study the majority of CGM and HCL users reported that they were willing to share real-time glucose information for timely support, incorporations of AI-based prediction of hypoglycemia [34] and adaptive tuning of bolus insulin parameters to prevent hyperglycemia [35] into mHealth could be considered as future research directions.

This study demonstrates that most advanced diabetes technology users are receptive to receiving mHealth support that could enhance their ability and motivation for effective self-care behaviors beyond the simple alarms for hypo- and hyperglycemia currently available via CGM systems and HCLs. Alarm fatigue can lead to turning off the hypo/hyperglycemia alarms or simply ignoring them [36]. Personalized interventions triggered by glucose levels could avoid alarm fatigue using tailored messaging supported by behavioral theories [37] for the generation of practical and culturally sensitive content [38].

We found that the majority of T1D advanced diabetes technology users were open to smartphone apps. However, a significant proportion also favored other communication channels such as SMS text messages and IVR calls, particularly those who were older. This finding underscores the significance of maintaining a diversity of mHealth approaches to promote intervention engagement in heterogeneous diabetes populations [21].

### Strengths and Limitations

This study is one of the first to report information related to the feasibility and potential interest in mHealth support among people with T1D using advanced diabetes technologies. Comparisons of characteristics of respondents to nonrespondents identified only a relatively small difference in the sex distribution, and analysis of survey data did not suggest that sex was related to any of the outcomes of interest. Glycemic indexes, including CGM glucose information, confirmed that both patient populations with and without controlled diabetes were receptive to receiving mHealth support.

Several limitations of this study should be considered. Participants were recruited from a population receiving care in a single tertiary academic health center. However, this health care system also has outreach clinics and medical services providing care to >1 million people in surrounding communities. The distribution of participants across racial/ethnicity groups and the proportion reporting use of an insulin pump were similar to the 2016-2018 T1D Exchange national report [6]. With the expanding use of smartphones in the United States [18,19] and increasing implementation of CGM systems [6], the findings are most applicable to tertiary health care centers and may be generalizable to other US T1D populations. Additionally, detailed information about the preferred features of mHealth apps, SMS text messages, and IVR intervention, including the timing and frequency of communication, were not collected. Future research should seek to deepen our understanding of these key dimensions of intervention design.

### Conclusions

We found that people with T1D using advanced diabetes technologies have access to mobile technologies and are receptive to receiving mHealth support for improving diabetes control. The majority of people in this population are open to smartphone apps or SMS text messages, and older individuals may favor SMS text messages or IVR calls for mHealth support.

## Abbreviations

AI: artificial intelligence
CGM: continuous glucose monitoring
EMR: electronic medical record
HbA_1c_: hemoglobin A_1c_
HCL: hybrid closed-loop insulin pump
IVR: interactive voice response
mHealth: mobile health
T1D: type 1 diabetes
TAR: time above range
TBR: time below range

## Data Availability

The data sets generated and analyzed during the study are available from the corresponding author on reasonable request.
